# Neck and back problems in adults with idiopathic scoliosis diagnosed in youth: an observational study of prevalence, change over a mean four year time period and comparison with a control group

**DOI:** 10.1186/s13013-017-0125-z

**Published:** 2017-06-08

**Authors:** Christos Topalis, Anna Grauers, Elias Diarbakerli, Aina Danielsson, Paul Gerdhem

**Affiliations:** 1 Department of Clinical Science, Intervention and Technology (CLINTEC), Karolinska Institutet, Stockholm, Sweden; 2 Department of Orthopaedics, Sundsvall and Härnösand County Hospital, Sundsvall, Sweden; 3 Department of Orthopaedics, Karolinska University Hospital, Stockholm, Sweden; 4 Department of Orthopaedics, Sahlgrenska University Hospital, Gothenburg, Sweden; 5 Department of Clinical Science, Intervention and Technology, Karolinska Institutet, K54, Karolinska University Hospital, SE-141 86 Stockholm, Sweden

**Keywords:** Idiopathic scoliosis, Neck pain, Back pain, Quality of life, Long-term outcome, 2

## Abstract

**Background:**

The knowledge is sparse concerning neck problems in patients with idiopathic scoliosis. This is an observational study including a control group which aims to describe the prevalence of neck problems and the association with back problems among adult individuals with and without idiopathic scoliosis.

**Methods:**

One thousand sixty-nine adults with a mean age of 40 years, diagnosed with idiopathic scoliosis in youth, answered a questionnaire on neck and back problems. Eight hundred seventy of these answered the same questionnaire at a second occasion in a mean of 4 years later. Comparisons were made with a cross-sectional population-based survey of 158 individuals. Statistical analyses were made with logistic regression or analysis of variance, adjusted for age, smoking status, and sex.

**Results:**

Individuals with scoliosis were previously untreated (*n* = 374), brace treated (*n* = 451), or surgically treated (*n* = 244). Of the individuals with scoliosis, 42% (*n* = 444) had neck problems compared to 20% (*n* = 32) of the controls (*p* = 0.001). The prevalence of neck problems was not affected by the type of treatment (*p* = 0.67) or onset of scoliosis; juvenile (*n* = 159) or adolescent (*n* = 910; *p* = 0.68). Neck and/or back problems were experienced by 72% of the individuals with scoliosis and 37% of the controls (*p* < 0.001). Of the individuals with scoliosis having neck problems, 81% also reported back problems, compared to 59% of the individuals in the control group (*p* < 0.001). The prevalence of neck and back problems was similar at the second survey.

**Conclusions:**

Neck problems are more prevalent and more often coexist with back problems in individuals with idiopathic scoliosis than in controls. The majority of individuals have persisting problems over time.

## Background

Idiopathic scoliosis is a three dimensional deformity of the spinal column that presents in otherwise healthy individuals. The prevalence of back problems as well as quality of life among adults with idiopathic scoliosis has been well described both in mid-term and long-term studies [1–5]. To the best of our knowledge, neck problems have not yet been under the focus in any study of idiopathic scoliosis. The prevalence of neck problems or pain has been described in a few studies. All have had some limitations, such as lack of a control group [6–8], or using a combined question for neck and back pain [9]. The relationship between regional cervical sagittal alignment and health-related quality of life in surgically treated individuals with adult spinal deformity has recently been reported, but the frequency of neck problems or pain was not described [6]. In addition, none of the previous studies have reported data for subgroups of idiopathic scoliosis patients such as men and individuals with a juvenile onset.

Hence, the aims of this study were to describe (i) the prevalence of neck problems in adults with and without idiopathic scoliosis diagnosed in youth and (ii) the relationship between neck and back problems, (iii) to analyze the effect of occupational strain and smoking habits on neck and back problems, and, finally, (iv) to describe any changes in the prevalence of neck problems over time in individuals with scoliosis.

## Methods

This is a multi-center observational study in adults diagnosed with either juvenile or adolescent idiopathic scoliosis, including comparisons with a cross-sectional population-based control group.

### Idiopathic scoliosis cohort

Individuals with juvenile idiopathic scoliosis (onset 4 to 9 years of age) or adolescent idiopathic scoliosis (onset 10 to 20 years of age), with a Cobb angle equal or greater than 10° were invited to take part in this survey [3, 10]. Age of scoliosis onset was based on self-reported data (in 94%) or according to the date of the first available radiograph which was confirmative for scoliosis (in 6%).

**Fig. 1:**
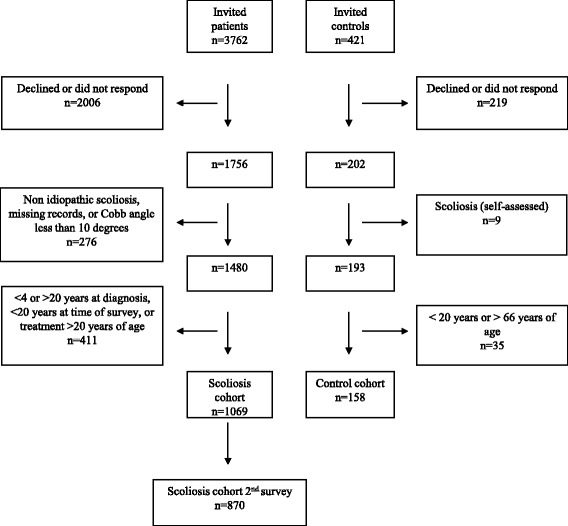
Flow chart of the study for the individuals with scoliosis and the controls

Recruitment took place from those currently under treatment or follow-up at the Karolinska University Hospital, Stockholm; the Skåne University Hospital, Malmö; and the Sundsvall and Härnösand County Hospital, Sundsvall, or from registers containing previously treated individuals at any of the three mentioned hospitals and from the Sahlgrenska University Hospital, Gothenburg. In this specific study, individuals under the age of 20 years or treated over the age of 20 years were excluded. A flow chart of the study is shown in Fig. 1.

In all, 1069 individuals with scoliosis completed the study. After a mean of 4 years (range 1–7) all individuals were asked to participate in a second survey, in which 870 (81%) individuals took part.

### Treatment

The individuals with scoliosis had been treated according to the general guidelines at the time of their treatment. Bracing was recommended at curves 24° to 50° in the 1960s and 1970s, except in lumbar curves, which were braced when Cobb angles were between 24° and 60°. For patients treated later, brace treatment was indicated in scoliosis curves between 25° and 45° in case of remaining growth. Larger curves after growth cessation resorted to surgery.

### Radiology and medical records

The radiological information was collected from the regular care of the individuals with scoliosis. The last radiograph was defined as the radiograph taken before the age of 27, since it was expected that all regular follow-ups had terminated at this age at all participating departments [3, 10].

### Individuals without scoliosis

A reference population was created by the Swedish Tax Agency by randomly selecting individuals from the Swedish population. Identical questionnaires as used for the patients, were mailed to 421 individuals, with up to three reminders, and 202 accepted to participate. After exclusion, 158 remained (Fig. 1). No clinical examination was done on the control cohort.

### Questionnaire

Identical questions regarding neck problems, back problems, work status, occupational strain, and smoking were used in both cohorts and at both surveys. The questions are listed in Appendix 1 [11].

### Statistics

Descriptive data are depicted as mean (SD) or number (%). Logistic regression or analysis of variance were used for statistical analyses and adjusted for age (20–44 vs. 45 years and older), smoking status, and sex (with the exception of analyses stratified on sex). The occupational strain data were dichotomized into two groups: sedentary/light and moderate/heavy occupational strain. Statistical software was IBM SPSS version 22. A *p* value less than 0.05 was considered statistically significant.

## Results

### Results of the first survey

Descriptive data for the 1069 individuals with scoliosis and the 158 controls are shown in Table 1.

**Table 1:** Descriptive data of the cohorts shown as number (%) or mean (SD). The scoliosis cohort is also divided into the different treatment groups

	Scoliosis				Controls
Variable	All (*n* = 1069)	Untreated (*n* = 374)	Brace treated (*n* = 451)	Surgically treated (*n* = 244)	(*n* = 158)
Age, years, first survey	41 (9)	40 (10)	40 (8)	43 (10)	45 (14)
Age, years, second survey^a^	45 (9)	44 (9)	45 (8)	46 (10)	–
Curve size, (°)^b^	28 (14)	23 (14)	30 (12)	30 (15)	–
Females	946 (88%)	320 (86%)	411 (91%)	215 (88%)	83 (53%)
Smokers	122 (11%)	53 (14%)	37 (8%)	32 (13%)	18 (11%)
Gainfully employed	931 (87%)	322 (86%)	404 (90%)	205 (84%)	124 (78%)
Moderate or heavy occupational strain^c^	246 (27%)	98 (31%)	91 (23%)	57 (28%)	42 (34%)

^a^Based on the 870 individuals with idiopathic scoliosis that answered to the second survey

^b^Curve size is defined as the Cobb angle of the largest curve, determined from the last available radiological follow-up before the age of 27. The curve size for men was 28° (17) and for women 27° (13), and for individuals with juvenile scoliosis 28° (14) and for patients with an adolescent scoliosis 28° (13). Curve apex was thoracic in 562, thoracolumbar in 172, lumbar in 105, and double primary in 230 cases. In the surgically treated, Harrington rods had been used in 213, segmental fixation in 28, and non-instrumented fusion in situ in 3 cases. A posterior approach had been used in 232 cases

^c^Answered by 924 individuals in the scoliosis group (321 untreated, 401 brace treated, and 202 surgically treated) and 124 individuals in the control group

The prevalence of neck problems, back problems and the co-existence of neck and back problems were more frequent among individuals with scoliosis than in those without (Table 2).

**Table 2:** Prevalence of neck problems and back problems in the 1069 individuals with idiopathic scoliosis and the 158 controls. Data is presented as number (%). The p﻿-value shown is for the comparison between the two groups, adjusted for age (20–44 or 45 years and older), smoking, and sex. The −2 log likelihood and Nagelkerke’s R﻿^2^﻿ for the model are shown

Variable	Scoliosis (*n* = 1069)	Controls (*n* = 158)	−2 log likelihood	Nagelkerke’s *R* ^2^	*p* value
Neck problems	444 (42%)	32 (20%)	1590	0.05	<0.001
Neck problems compromising the level of activity^a^	187 (42%)	9 (28%)	631	0.04	0.11
Back problems	688 (64%)	46 (29%)	1560	0.10	<0.001
Neck and back problems	362 (34%)	19 (12%)	1460	0.07	<0.001
Neck or back problems	770 (72%)	59 (37%)	1453	0.10	<0.001

^a^Answered by 444 individuals in the scoliosis group and 32 in the non-scoliosis group

Neck problems compromising the level of activity were more frequent in the scoliosis population, but the difference did not reach statistical significance (Table 2).

Neither the prevalence of neck and/or back problems nor the prevalence of neck problems compromising the activity level were affected by the type of previously performed treatment for the scoliosis curve (no treatment/bracing/surgery) (Table 3).

**Table 3:** There were no differences in the prevalence of neck and/or back problems between untreated, brace-treated, or surgically treated individuals. Data are shown as numbers (%). P-values are shown for the comparison between the three groups, adjusted for age (20–44 and 45 years and older), smoking, and sex. The F test and R^2^ for the model are shown

	Scoliosis					
Variable	Untreated (*n* = 374)	Brace treated (*n* = 451)	Surgically treated (*n* = 244)	*F*	*R* ^2^	*p* value
Neck problems	150 (40%)	193 (42%)	101 (41%)	3.7	0.02	0.67
Neck problems compromising the level of activity^a^	67 (45%)	75 (39%)	45 (45%)	2.7	0.03	0.78
Back problems	258 (69%)	274 (61%)	156 (64%)	6.5	0.03	0.06
Neck and back problems	133 (36%)	146 (32%)	83 (34%)	5.0	0.02	0.67
Neck or back problems	275 (73%)	321 (71%)	174 (71%)	4.9	0.02	0.61

^a^Answered by 150 individuals in the untreated group, 193 in the brace-treated group and 101 in the surgically treated group

**Fig. 2:**
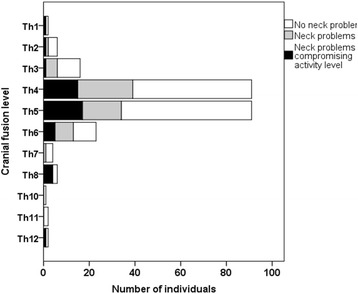
The proportion of patients experiencing neck problems or neck problems affecting the activity level was not related to the cranial extent of the fusion (*p* = 0.72 and *p* = 0.10, respectively), both analyses adjusted for age (20–44 vs. 45 years and older) smoking, and sex. There were no patients that were fused to the ninth thoracic vertebra

In the surgically treated individuals, neck problems and activity level were not related to the cranial fusion level (Fig. 2).

Forty-three percent of the women in the scoliosis group had neck problems compared to 22% of the women in the non-scoliosis group (*p* < 0.001). Corresponding figures for men were 32% and 19% (*p* = 0.029). Women with scoliosis had a higher prevalence of neck problems than men with scoliosis (43% vs. 32%, *p* = 0.030).

Comparisons between patients with adolescent (*n* = 910) and juvenile onset (*n* = 159) scoliosis showed no differences for the prevalence of neck problems in general or for neck problems compromising the activity level (*p* = 0.68 and *p* = 0.34, respectively).

In the scoliosis group, those with moderate and heavy occupational strain had a higher prevalence of neck problems compromising the activity level than those with sedentary and light work (*p* = 0.047), while no differences were found within the control group (*p* ≥ 0.10).

There were also significantly more smokers in the group with scoliosis who had neck problems (54%) compared to the non-smokers (40%) (*p* = 0.004). Corresponding figures for the non-scoliosis group was 4 out of 18 (22%) and 28 out of 140 (20%) (*p* = 0.72).

### Results of the second survey

Out of the 870 individuals with scoliosis that answered the second questionnaire, 367 (42%) of these reported neck problems. Of these 367 individuals, 267 (73%) had reported neck problems also at the first survey. Back problems were reported by 524 (60%) of the 870 individuals that answered the second questionnaire. Of these 524 individuals, 460 (88%) reported back problems also at the first survey.

### Non-response analysis

We compared the 199 individuals with scoliosis that did not respond to the second survey with the 870 individuals who did respond. There were no differences in the prevalence of back problems (*p* = 0.4) or neck problems (*p* = 0.5) in the first survey between the 199 non-responders and the 870 responders. Differences were seen for age, sex, and smoking; the mean age among responders was 41 years, compared to 39 years among non-responders (*p* = 0.003); 89% of the responders were females, compared to 84% of the non-responders (*p* = 0.046); and 90% of the responders were non-smokers, compared to 81% of the non-responders (*p* = 0.001).

## Discussion

In summary, neck problems are more common and more often coexist with back problems in individuals with idiopathic scoliosis than in the general population.

Previous studies on neck problems in idiopathic scoliosis patients show contradictory results. In a follow-up 27 years after non-instrumented fusion for idiopathic scoliosis, 14 out of 22 (64%) patients complained of neck pain [7]. Another study reported significantly less neck pain but more generalized back pain in adult subjects with idiopathic scoliosis noted after age nine who were non-operated than in controls [9]. However, the participants in that study had a limited amount of answer alternatives and had to choose between neck pain, upper back pain, lower back pain, or generalized pain only, leading perhaps to some uncertainty about the true prevalence of neck pain alone or combined with other regions in the spinal column.

Edgar and Mehta found that cervicodorsal pain was more common after non-instrumented fusion for idiopathic scoliosis than after conservative treatment about 15 years after reaching skeletal maturity [8]. In more recent studies on both braced and operated patients with adolescent idiopathic scoliosis, neck pain was not significantly more common among the scoliosis patients than in a matched control group, 17% of the braced and 27% of the operated patients compared to 17% of the controls [1, 2]. It was also found that neck pain was significantly less usual in patients that were fused more cranially, 14% of those fused to the T4 or above admitted neck pain compared to 35% in those fused to the T5 only or below.

In this study, we could not confirm that surgical treatment was associated with more neck problems. A distinct favor of the current study is its size, which is considerably larger than previous studies, giving better precision of estimates.

The caudal fusion level may affect activity level [3]. We therefore analyzed whether the cranial fusion level had the same effect on neck activity. No such association could be found. Therefore, the choice of the upper level of the thoracic fusion does not seem to affect the activity level.

We could not find any differences related to juvenile or adolescent onset, similar to previous reports on back problems [3, 12]. It seems evident that at least for individuals that are treated according to the general guidelines, age of diagnosis is not important for the long-term outcome concerning neck or back problems. That women experience slightly more neck problems than men seems to be consistent with previous reports [13].

The co-existence of chronic neck and back pain is common [14, 15]. Population-based data from Sweden, with individuals of similar ages as in this study, show similar prevalence of chronic neck pain [16] and co-existence of neck and back pain as in our data of neck and back problems in the controls [15].

Smokers more often reported neck problems than non-smokers in the scoliosis group, but this was not seen in the control group. This comparison is somewhat hampered by the low amount of smokers and the group size of the controls. The relationship between smoking and neck pain and problems is conflicting [13, 16].

The study design has advantages such as a sufficient group size enabling comparisons between treatment groups, individuals with juvenile and adolescent onset, and males and females. It also includes a prospective observation indicating changes over time. In addition, the inclusion of a control group increases the validity of the findings.

One might argue that physical activity can compensate pain issues and problems in general in scoliosis patients. However, data based on the same individuals as in this study indicate that self-reported physical activity does not seem to differ between individuals with and without scoliosis [17] despite the apparent differences in the prevalence of neck and back problems.

However, this study also has some limitations that have to be discussed. The main limitations include the response rate and the use of non-validated questions, including the use of the term neck problems rather than neck pain. Moreover, extra radiographic surveillance at the time of the study was not available; thus, any association between coronal and sagittal plane parameters and the questionnaire data could therefore not be determined.

A major limitation of this study is the response rate, 47% in the scoliosis patients and 48% in the controls. We have no information on the initial non-responders and cannot be certain on the representativeness of the available sample. However, representativeness is not necessary when interpreting the relationships between neck problems and other variables [18]. Another major limitation is the lack of information on whether the neck pain was acute, sub-acute, or chronic, which may have different impacts on quality of life and lead to different therapeutic approaches. The higher prevalence of neck problems in the scoliosis group does not seem to have an impact on the strain and ability to work [3].

For the second survey in the scoliosis cohort, a non-response analysis indicated that the prevalence of neck and back problems did not differ between responders and non-responders, and the differences in other descriptive variables were small.

The today widely used and validated Scoliosis Research Society (SRS)-questionnaire was not available in Swedish at the time of the study start [19], and anyhow, it does not address neck problems. Questionnaires specifically studying neck pain in scoliosis patients are not available, but others such as the Neck Disability Index would have been an option [20]. Nevertheless, the questions used were simple and straightforward and both the scoliosis group and the controls answered to exactly the same questions, to some extent making up for the use of previously non-validated questions.

We deliberately chose the term neck problems, embracing pain and signs and symptoms manifesting in the cervical area such as discomfort, stiffness, soreness, and numbness, giving a wider view of any problem in the neck. The study design was influenced by the fact that studies focused on neck pain or other neck problems did not exist for idiopathic scoliosis patients. To date, this study is the only one that elucidates these problems to such an extent. It includes an adult population with a span of 20 to 65 years of age, all treated before the age of 20, indicating the long-term results and the natural history of neck problems in the idiopathic scoliosis population.

There were some differences in age and sex distribution between the scoliosis and control groups. The control group was deliberately sampled with a similar proportion of men and women to be able to study men separately with a smaller control group, than if sex had been matched to equal proportions with the scoliosis cohort. Age spans in the groups were identical and mean ages differed only slightly. Both sex and age differences were accounted for in the calculations. It is possible that other unmeasured confounders could have a role.

The controls answered only once to the survey, and therefore lacking prospective data. However, other studies indicate that the prognosis of neck problems in the scoliosis population does not diverge from the expected in the general population [13].

## Conclusions

The current study and a recent increase in the interest of the cervical spine and its relation to health-related quality of life [6] may intensify further studies in this area.

In summary, neck problems are more prevalent and more often coexist with back problems in individuals with idiopathic scoliosis than in controls. The majority of individuals seem to have similar problems some years later.

## Appendix

Self-assessment questionnaire

1. Do you experience any neck problems?
  I. Yes
  II. No
2. Do your neck problems compromise your activity level?
  I. Yes
  II. No
3. Do you experience any back problems?
  I. Yes
  II. No
4. Do your back problems compromise your activity level?
  I. Yes
  II. No
5. Are you gainfully employed?
  I. Yes
  II. No
6. Which of the following categories describe your activity level at work (including household work)?
  I. Mostly sedentary work
  II. Light work with walking to some extent and no heavy lifting
  III. Moderately heavy work with a lot of walking as well as lifting
  IV. Heavy work
7. Do you smoke?
  I. Yes
  II. No
